# Outcome of small cell lung cancer (SCLC) patients with brain metastases in a routine clinical setting

**DOI:** 10.2478/v10019-012-0007-1

**Published:** 2012-01-02

**Authors:** Mirko Lekic, Viljem Kovac, Nadja Triller, Lea Knez, Aleksander Sadikov, Tanja Cufer

**Affiliations:** 1 University Clinic for Respiratory and Allergic Diseases Golnik, Slovenia; 2 Institute of Oncology Ljubljana, Ljubljana, Slovenia; 3 University of Ljubljana, Faculty of Computer and Information Science, Slovenia

**Keywords:** small-cell lung cancer, brain metastases, prophylactic cranial irradiation

## Abstract

**Background:**

Small cell lung cancer (SCLC) represents approximately 13 to 18% of all lung cancers. It is the most aggressive among lung cancers, mostly presented at an advanced stage, with median survival rates of 10 to12 months in patients treated with standard chemotherapy and radiotherapy. In approximately 15-20% of patients brain metastases are present already at the time of primary diagnosis; however, it is unclear how much it influences the outcome of disease according the other metastatic localisation. The objective of this analysis was to evaluate the median survival of SCLC patients treated by specific therapy (chemotherapy and/or radiotherapy) with regard to the presence or absence of brain metastases at the time of diagnosis.

**Patients and methods:**

All SCLC patients have been treated in a routine clinical practice and followed up at the University Clinic Golnik in Slovenia. In the retrospective study the medical files from 2002 to 2007 were review. All patients with cytological or histological confirmed disease and eligible for specific oncological treatment were included in the study. They have been treated according to the guidelines valid at the time. Chemotherapy and regular followed-up were carried out at the University Clinic Golnik and radiotherapy at the Institute of Oncology Ljubljana.

**Results:**

We found 251 patients eligible for the study. The median age of them was 65 years, majority were male (67%), smokers or ex-smokers (98%), with performance status 0 to 1 (83%). At the time of diagnosis no metastases were found in 64 patients (25.5%) and metastases outside the brain were presented in 153 (61.0%). Brain metastases, confirmed by a CT scan, were present in 34 patients (13.5%), most of them had also metastases at other localisations. All patients received chemotherapy and all patients with confirmed brain metastases received whole brain irradiation (WBRT). The radiotherapy with radical dose at primary tumour was delivered to 27 patients with limited disease and they got 4–6 cycles of chemotherapy. Median overall survival (OS) of 34 patients with brain metastases was 9 months (95% CI 6–12) while OS of 153 patients with metastases in other locations was 11 months (95% CI 10–12); the difference did not reach the level of significance (p = 0.62). As expected, the OS of patients without metastases at the time of primary diagnosis turned out to be significantly better compared to the survival of patients with either brain or other location metastases at the primary diagnosis (15 months *vs* 9 and 11 months, respectively, p < 0.001).

**Conclusions:**

In our investigated population, the prognosis of patients with extensive SCLS with brain metastases at the primary diagnosis treated with chemotherapy and WBRT was not significantly worse compared to the prognosis of patients with extensive SCLC and metastases outside the brain. In extensive SCLC brain metastases were not a negative prognostic factor *per se* if the patients were able to be treated appropriately. However, the survival rates of extensive SCLC with or without brain metastases remained poor and novel treatment approaches are needed. The major strength of this study is that it has been done on a population of patients treated in a routine clinical setting.

## Introduction

Small cell lung cancer (SCLC) is a life-threatening disease, typically caused by cigarette smoking. It is almost exclusively seen in current or former smokers. SCLC represents approximately 13–18% of all lung cancers with a varying incidence in different countries.^1^ The incidence of SCLC is in decrease and the ratio of incidence between SCLC/NSCLC is decreasing as well. Without treatment, this cancer has the most aggressive clinical course of all lung cancer types, with survival rates between 2 to 4 months.

A recommendation to use TNM International Union Against Cancer (UICC) staging system for all SCLC cases was regularly accepted.^2^–^4^ However, a simple two-stage system developed by the Veteran’s Administration Lung Cancer Study Group as limited (stage) disease (LD) or extensive (stage) disease (ED) was more frequently used in clinical practice.^5^ Patients with limited disease have the involvement restricted to one hemithorax that can be encompassed within a tolerable radiation field. Extensive disease is defined as disease beyond one hemithorax and may include malignant pleural or pericardial effusion or haematogenous metastases.

At the time of diagnosis approximately 70% of patients have ED. Patients with extensive-stage SCLC are considered incurable, and have median survival time of approximately 9–10 months from diagnosis with the current standard treatment.^5^ Patients with limited SCLC have a median survival of approximately 18 months and can be cured. By using multimodality therapy, including concurrent chemotherapy and radiotherapy, long-term disease-free survival rates can be achieved in approximately 20–25% of patients.^6^ Although SCLC is regarded as highly sensitive to both chemotherapy and radiotherapy, fast development of resistance is a major problem. Only a modest improvement in survival has been achieved during the last 20 years, and prognosis remains poor with 5-year survival rates of around 10% and 2% in LD and ED, respectively.^7^

At the time of the initial diagnosis, approx. 20% of the patients with SCLC have detectable brain metastases, and the incidence of such metastases increases considerably during the course of disease, approaching 80% at 2 years. The standard treatment of clinically evident brain metastases is whole brain radiotherapy (WBRT), usually in combination with corticosteroids and the solitary metastasis might be additional irradiated with stereotactic radiotherapy or Gamma Knife.^8^–^10^ The response rate to brain irradiation is around 50%, but survival rates are relatively short. In a majority of observations published until now brain metastases were found to be an independent prognostic factor for short survival. However, few individual trials have not found brain metastases to affect survival adversely.^11^,^12^,^13^ In one of these trials the survival rates of the patients with extensive disease with or without brain metastases at the time of diagnosis was done. In this particular study extensive-stage patients with brain metastases and additional sites of metastatic disease had shorter survival compared to those with brain metastases alone (5 vs. 11 months).^11^ There are few studies showing comparable survival rates in patients with only brain metastases to the survival rates achieved in patients with LD.^12^,^13^

The objective of this retrospective study was to evaluate the median survival of SCLC patients with or without brain metastases at the time of diagnosis treated in a routine clinical practice before the introduction of PCI as a standard treatment procedure.

## Patients and methods

Patients with SCLC diagnosed and treated at the University Clinic Golnik between 2002 to 2007, with performance status ≤2 and eligible for standard treatment of chemotherapy +/− radiotherapy were included in retrospective study. Baseline evaluations included medical history and a radiological examination. In all patients chest X-ray, CT scans of the chest, abdomen and brain were performed.^3^ Additional exams, like bone scintigraphy, were performed only if symptoms were present. Pathological confirmation of SCLC was mandatory.^14^ Patients were treated according to the guidelines valid at that time. All patients with radiologically confirmed brain metastases, with or without neurological symptoms, received whole brain irradiation (WBRT). The radiotherapy was applied at the Institute of Oncology Ljubljana, Slovenia, and patients were followed-up regularly at the University Clinic Golnik.

### Statistical analysis and ethical consideration

The endpoint in this study was overall survival (OS) which was calculated from the date of diagnosis to the date of death from any cause, or the date of the last follow-up. OS as a function of the location of metastases was estimated by the Kaplan-Meier method, and the log-rank test was used to test the differences. Computations were done with the SPSS 16 statistical package. All reported p-values are two-tailed.

The study was carried out according to the Declaration of Helsinki.

## Results

### Patients

In the study, 251 patients were included. Patients’ characteristics are presented in Table 1. Median age was 65 years, majority of them were male (67%), smokers or ex-smokers (98%), with performance status from 0 to 1 (83%). At the time of diagnosis, no metastases were found in 64/251 (25.5%) patients, metastases outside the brain were present in 153/251 (61.0%) patients, while brain metastases, confirmed by a CT scan, were present in 34/251 (13.5%) patients. In 8 out of 34 patients, brain metastases were the only site of an extensive disease.

Brain metastases at diagnosis had 34 patients. All of them received whole brain irradiation (WBRT). They were irradiated with ^60^Co source or at linear accelerator with x-rays and high energy of 5 or 6 MeV. The most frequent tumour doses were 30 Gy in 10 fractions, 30 Gy in 12 fractions or 20 Gy in 5 fractions (Table 2). Other regimes of radiotherapy were less frequent. Seventeen out of 64 patients with LD underwent prophylactic brain irradiation at that time. They represented only 6.8% of whole 251 patients.

Twenty-six (10.4%) patients with LD were also treated with thoracic irradiation. A radical tumour dose was applied and 4–6 cycles of chemotherapy.

All patients received chemotherapy, 177/251 (70.5%) with etoposide and platinum (EP), 39/251 (15.5%) with cyclophosphamide, doxorubicin, vincristine (CEV) and 35/251 (13.9) with CEV-EP (Table 3).

### Survival

**FIGURE 1:**
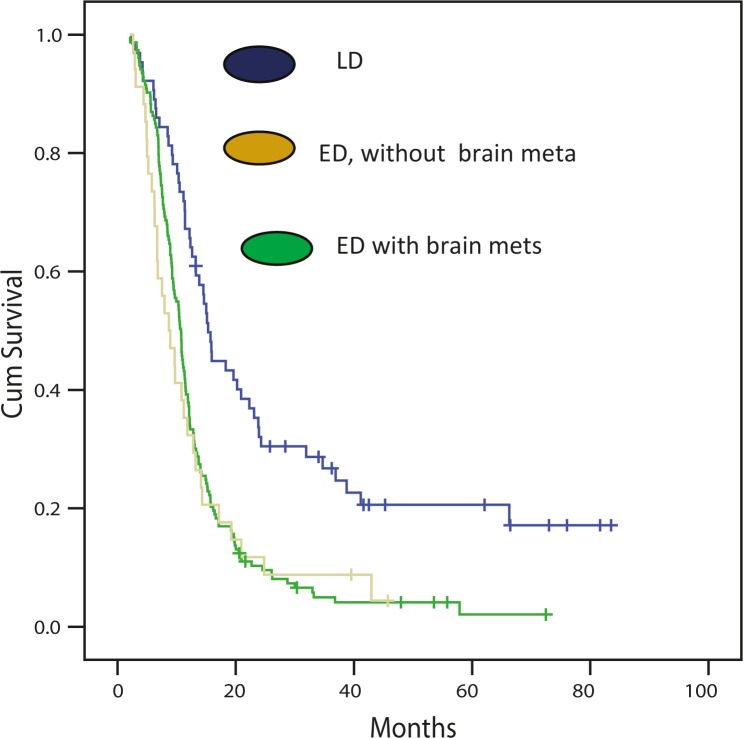
Survival of patients with limited stage disease (LD), extensive stage disease (ED) and according the brain metastases.

No statistical significantly difference in OS between ED patients with and without brain metastases at the time of diagnosis was observed (p = 0.62). Median OS of 34 patients with brain metastases was 9 months (95% CI, 6 to 12) while median OS of 153 patients with metastases in other locations at primary diagnosis was 11 months (95% CI, 10 to 12). As expected, the OS of patients with limited disease was significantly better compared to the survival of patients with either brain or other location metastases at the primary diagnosis (15 months *vs* 9 and 11 months, respectively, p < 0.001) (Figure 1).

The number of patients with brain metastases as the only site of metastases, was too small (n = 8) to allow a separate analysis.

## Discussion

The prognosis of patients with SCLC and brain metastases at diagnosis is poor. Nevertheless, our observations made in a substantially large population of 251 SCLC patients, who were suitable for standard specific therapy, *i.e.* chemotherapy and WBRT if necessary, did not confirm worse survival of ED patients with brain metastases compared to those without brain metastases at the time of diagnosis. In our study, LD patients had significantly better median survival than ED patients (15 vs. 11 months). However, there was no significant difference between the median survival of extensive stage patients with brain metastases at initial presentation and median survival of extensive stage patients without brain metastases (9 vs. 11 months).

Similar median survival (11 months) for extensive stage patients without brain metastases at the time of diagnosis was reported by Crane *et al*.^15^ however in this trial, with 153 patients included, survival of extensive stage patients with brain metastases at the time of diagnosis was only 6 months, significantly worse compared to the survival of extensive stage patients without brain metastases at the time of diagnosis. The difference between our observation and that by Crane *et al*. might be due to the relatively small number of patients with brain metastases in both trials as well as due to possible differences in other patients’ characteristics. It is well known that the extent of the disease, characterized by performance status, LDH levels and the number of metastatic sites, determines the prognosis of ED even more than the site of metastases.^16^–^19^ The percentage of patients with brain metastases in our collective of patients was rather low (13.5%) which might be the consequence of our selection criteria, *i.e.* performance status equal or less than 2. Performance status was found to be correlated with the extent of disease as well as CNS involvement.^17^,^19^

As it was clearly shown by a large pooled analysis done by Hazel *et al*.^12^ and confirmed later by Giannone *et al*.^11^ through a large retrospective observation with more than four hundred patients included, brain metastases alone without the additional burden of extensive disease outside the brain actually do not mean a bad prognosis at all. In these two trials the survival rates of patients with a small burden of disease, *i.e.* brain metastases alone, were actually comparable to the survival rates of patients with limited disease. The similar conclusion was done by Kochhar *et al.* although they retrospectively reviewed only 30 patients.^20^ Unfortunately, our group of patients with brain metastases as the only site of metastases, was too small to allow a separate analysis.

Yet another reason for comparable survival rates of our ED patients with or without brain metastases might be that our entire patient received chemotherapy in addition to WBRT. The selection of our patients was actually made according to their suitability for specific therapy and PS ≤ 2.

Prophylactic cranial irradiation (PCI) has been explored since the 1970s as a therapeutic option that could lower the rates of brain relapse and possibly increase patients’ survival because it is well known that the central nervous system is relatively refractory to chemotherapy due to the blood-brain barrier. PCI was found to improve survival rates significantly not only in limited stage but also in extensive stage SCLC responding to chemotherapy, and is now recommended as a part of the standard treatment patients with LD and ED SCLC.^19^,^21^–^27^ The first meta-analysis by Auperin *et al*. in 1999 reported the 5.4% increase in the rate of survival at three years for patients achieving complete response on primary chemoradiation therapy.^24^ It was followed by a large EORTC (European Organisation for Research and Treatment of Cancer) trial that confirmed significant benefit of PCI in all patients achieving response (complete or partial) to first-line systemic therapy.^25^ Based on these observations PCI represents a standard treatment approach nowadays, which ultimately results in a lower rate of brain metastases diagnosed during the course of the disease.^19^ However, the percentage of patients presenting with brain metastases at the time of diagnosis remains stable, *i.e*. between 14–20%. Our group of patients with PCI, was (similar as group with brain metastases alone) too small to allow a separate analysis.

The question remains on optimal treatment of brain metastases in SCLC. For extensive central nerve system involvement the treatment of choice is WBRT, however the optimal treatment strategy for a solitary brain metastasis, which is quite rare in SCLC, has still not been defined. It is known for some other cancers, such as non-small cell lung cancer and breast cancer, that surgical or stereotactic irradiation of solitary brain metastasis followed by WBRT gains better survival rates compared to WBRT alone.^9^,^10^ Despite the lack of scientific evidence based on randomized trials such an approach may be superior in selected SCLC patients as well. Novel treatment approaches incorporating new irradiation techniques and novel drugs that might cross blood-brain barrier are urgently needed to change the faith of this still deadly disease. Several targeted agents have been introduced into clinical trials in SCLC, including matrix metalloproteinase inhibitors, thalidomide, and vaccines, with negative results being more commonly reported than positive ones so far. Although initial attempts at targeted therapy in SCLC have been unsuccessful, several newly identified targets agents, such as anti-angiogenic strategies and Bcl-2 inhibitors, hold promise and are being tested in the ongoing clinical trials.^28^–^30^

In conclusion, the prognosis of patients with extensive SCLS with brain metastases at the time of the diagnosis, suitable for specific chemotherapy and radiotherapy, does not seem to be significantly worse compared to the prognosis of patients with extensive SCLC without brain metastases. In extensive SCLC brain metastases might not represent a negative prognostic factor *per se* if they are treated appropriately. However the survival rates of these patients was still dismal with median survival rate of 9 months observed in our study. To further improve the prognosis of SCLC patients, the incorporation of new treatment strategies, such as stereotactic irradiation and invent of novel therapies, seems to be mandatory.

## Figures and Tables

**TABLE 1:** Patients’ characteristics (n = 251)

	**No of patients**	**%**
**GENDER**		
Male	170	68.0
Female	81	32.0
**AGE**		
< 70	163	65.0
≥ 70	88	35.0
**SMOKING STATUS**		
Current smoker	142	57.0
Never smoker	2	0.8
Ex-smoker	97	38.2
Unknown	10	4.0
**STAGE**		
LD	64	25.5
ED		
- With brain metastases	34	13.5
- Without brain metastases	153	61.0
**PERFORMANCE STATUS**		
PS-0	43	17.0
PS-1	166	66.0
PS-2	42	17.0

**TABLE 2:** Whole brain irradiation (WBRT) of 34 patients with brain metastases at diagnosis

**Tumour dose**	**Modality**	**Frequency**	**%**
30 Gy in 10 fractions	^60^Co	8	23.5
	Linear accelerator	1	2.9
30 Gy in 12 fractions	^60^Co	2	5.9
	Linear accelerator	4	11.8
*20 Gy in 5 fractions*	^60^Co	6	17.6
	Linear accelerator	1	2.9
Other regime	^60^Co	6	17.6
	Linear accelerator	6	17.6
TOTAL		34	100.0

**TABLE 3:** Chemotherapeutic schemes of 251 patients

	**Frequency**	**%**
EP	177	70.5
CEV	39	15.5
EP + CEV	35	13.9
TOTAL	251	100.0

EP = etoposide and platinum;

CEV = cyclophosphamide, doxorubicin, vincristine
